# Kick Control: Using the Attracting States Arising Within the Sensorimotor Loop of Self-Organized Robots as Motor Primitives

**DOI:** 10.3389/fnbot.2018.00040

**Published:** 2018-07-11

**Authors:** Bulcsú Sándor, Michael Nowak, Tim Koglin, Laura Martin, Claudius Gros

**Affiliations:** ^1^Department of Physics, Babes-Bolyai University, Cluj-Napoca, Romania; ^2^Institute for Theoretical Physics, Goethe University Frankfurt, Frankfurt am Main, Germany

**Keywords:** closed-loop robots, limit cycles, sensorimotor loop, self-organized locomotion, compliant robot, robophysics

## Abstract

Self-organized robots may develop attracting states within the sensorimotor loop, that is within the phase space of neural activity, body and environmental variables. Fixpoints, limit cycles and chaotic attractors correspond in this setting to a non-moving robot, to directed, and to irregular locomotion respectively. Short higher-order control commands may hence be used to kick the system from one self-organized attractor robustly into the basin of attraction of a different attractor, a concept termed here as kick control. The individual sensorimotor states serve in this context as highly compliant motor primitives. We study different implementations of kick control for the case of simulated and real-world wheeled robots, for which the dynamics of the distinct wheels is generated independently by local feedback loops. The feedback loops are mediated by rate-encoding neurons disposing exclusively of propriosensoric inputs in terms of projections of the actual rotational angle of the wheel. The changes of the neural activity are then transmitted into a rotational motion by a simulated transmission rod akin to the transmission rods used for steam locomotives. We find that the self-organized attractor landscape may be morphed both by higher-level control signals, in the spirit of kick control, and by interacting with the environment. Bumping against a wall destroys the limit cycle corresponding to forward motion, with the consequence that the dynamical variables are then attracted in phase space by the limit cycle corresponding to backward moving. The robot, which does not dispose of any distance or contact sensors, hence reverses direction autonomously.

## 1. Introduction

The sensorimotor system is in general a product of evolution, development, learning, and adaptation (Todorov, 2004). One may examine alternatively whether self-organizing principles (Prokopenko et al., 2009) are capable to generate locomotion, in particular for the case of embodied (Ghazi-Zahedi et al., 2017) and/or biologically inspired robots (Pfeifer et al., 2007). Self-organization may serve in this context to generate a palette of behavioral primitives (Tani and Ito, 2003), or, on a higher level, to generate complex and playful behavior (Martius et al., 2013).

Attracting states in the sensorimotor loops corresponding to regular and exploratory motion, that is respectively to limit cycles and chaotic attractors, can be generated following two complementary routes. Within the first approach, which is especially suited for settings involving a large number of degrees of freedom (Kubisch et al., 2011), the optimal mapping between sensors and actuators is learned. Learning is on the other side absent when generative principles are implemented and studied (Gros, 2014). Short-term synaptic plasticity, a transient form of mostly presynaptic neural plasticity (Hennig, 2013), has been shown in this context to generate limit cycles (Toutounji and Pasemann, 2014) and chaotic attractors (Martin et al., 2016). Other examples are the homeostatic principles regulating the average neural activity (Linkerhand and Gros, 2013), which have been shown to induce surprisingly complex locomotive patters (Sándor et al., 2015).

The control of wheeled robots, e.g., with skid steering (Kozłowski and Pazderski, 2004), is well established, with explicit mathematical models (Das et al., 2006) being often the basis for the regulation of either the angular velocity of the individual wheels (Jimenez-Fernandez et al., 2012), or of the respective torque (Mandow et al., 2007). One may obtain the sensorimotor mapping relevant for the neuromorphic robot at hand also by training large neural networks (Conradt et al., 2015). As an alternative, we consider here exceedingly simple neural control schemes that are based on physical principles and not on adaptive learning. Typically, we need just one or two neurons per actuator.

Our starting point is the observation that neural activity, such as the spiking rate *y*, has a defined but limited range, say *y* ∈ [0, 1]. The activity of an output motor neuron could therefore be mapped, in principle, directly to the target angular velocity ω of the wheel, e.g., via ω ~ (2*y* − 1). Forward and backward motions would correspond in this setting to distinct neural activity patterns. We examine here in contrast a neural controller for which the time reversal symmetry between forward- and backward motion is broken spontaneously under the influence of initial conditions.

Our robots are equipped with two active wheels and a third passive support wheel. A maximum of two neurons per wheel generate self-organized locomotion, which is both compliant and variable. A simulated transmission rod is used to map the forth-and-back motion of the neural activity level *y* ∈ [0, 1] to the motor command in a manner that mirrors the transmission mechanism used by traditional steam locomotives to transmit the force generated by the pressurized steam piston to the rotating wheel.

Both computer simulations and experiments with real robots are performed in order to assess the feasibility of the proposed control mechanism. We find that locomotion is generated robustly both for individual robots and for trains of passively coupled two-wheeled cars. The only sensory information driving the neural activity is propriosensoric, namely the current angle of the wheel the neuron controls. Cross-wheel information exchange is absent. Highly complex behavioral patterns (such as forward and backward locomotion, exploratory chaotic motion) emerge nevertheless upon interaction with the environment, which modifies the attractor landscapes of the individual wheels.

Experimenting with additional top-down control signals we find that it is possible to kick individual wheels from one attractor into the basin of attraction of another attracting state. The self-organized limit cycles and chaotic attractors forming in the sensorimotor loop may hence be used also as motor primitives.

The rest of the paper is structured as follows. In section 2, the concept of kick control is introduced and defined mathematically, followed by the description of the proposed controller and of the experimental setup. In section 3, three possible implementations of kick control and their reliability tests are presented as a proof of concept. The experimental findings are then investigated via a simple analytic model as well. Finally, a summary is given in section 5.

## 2. Materials and methods

From a general perspective we are interested in attracting states that form in the sensorimotor loop. Defining with **x**_*R*_ and **x**_*E*_ the dynamical variables of the robot (R) and of the environment (E) we have

(1) $$\begin{array}{l} {\overset{\cdot }{\text{x}}}_{R}={\text{f}}_{R}\left({\text{x}}_{R},{\text{x}}_{E};{\text{P}}_{R}\right),\;{\overset{\cdot }{\text{x}}}_{E}={\text{f}}_{E}\left({\text{x}}_{R},{\text{x}}_{E};{\text{P}}_{E}\right) \end{array}$$

for the combined dynamics, where **P**_*R*_ and **P**_*E*_ parametrize respectively the time evolution of the robot and of the environment. Parameters distinguish themselves in our notation from variables in the respect that they change either only very slowly, as the result of a separation of time scales, or via actions that can be considered external. Control signals will modify the evolution equations (1), which describe as such locomotion generated autonomously within the sensorimotor loop.

### 2.1. Kick control

Locomotion is characterized typically by timescales of seconds. One speaks of “kick control,” when a robot is subjected to control sequences that are shorter than the time needed to complete a movement, e.g., of the order of 50–200 ms. Kick control is functionally dependent on the existence of multiple attracting states in the sensorimotor loop that correspond to distinct locomotive patterns. The control signal then serves “to kick” the dynamical system (1) into the basin of attraction of the desired attracting state. There are two mutually not excluding venues.

– “Frozen” kick control is present when the control pulse Δ**x**_*R*_ acts via
  (2) $$\begin{array}{l} {\text{x}}_{R}\;\to \;{\text{x}}_{R}+\Delta {\text{x}}_{R} \end{array}$$
  exclusively onto the variables **x**_*R*_ of the robot. The parameters **P**_*R*_ are not changed, they remain frozen. The state of the system is kicked here from its present state **x**_*R*_ to a new state, viz to ${\text{x}}_{R}^{\prime }={\text{x}}_{R}+\Delta {\text{x}}_{R}$. A sudden change of parameters as in (2) corresponds to an additional strong temporary force within the right-hand-side of the corresponding Newton equation of motion (i.e., to a kick).
– “Quenched” kick control is realized when the control pulse Δ**P**_*R*_(*t*), which may be something like a rectangular pulse or a broadened δ-function, leads via
  (3) $$\begin{array}{l} {\text{P}}_{R}\;\to \;{\text{P}}_{R}+\Delta {\text{P}}_{R}\left(t\right) \end{array}$$
  to a sudden but transient change of the parameters **P**_*R*_ of the robot. The near instantaneous change of parameters catapults the system into a quenched configuration for which the attracting states of evolution equations (1) are morphed.

Quenched kick control will need in general a somewhat longer control signal. The reason is that the time evolution under the influence of the morphed attractors, that are present over the duration of the control pulse for the case of quenched kick control, needs to progress to a point in which the system finds itself within the basin of attraction of a different attractor once the control pulse ceases.

We have defined control signals as changes of either the variables or of the parameters of the robot. We may consider alternative events that lead to changes of the state of the environment, notably of the parameters **P**_*E*_. This happens in particular when the robot interacts with other objects, e.g., when it bounces against a wall. The resulting transition to a different attracting state may hence also be described at times within the terminology of kick control.

### 2.2. Simulated steam-locomotive actuator

We consider robots for which the active wheels are controlled independently by simple proprioceptual rate-encoding neurons. A finite angular velocity is attained when the internally generated torque interacts with the external response resulting from friction forces, gravity and inertia.

Neural activity covers a finite range, which can be normalized to the interval [0, 1]. A straightforward route for translating the neural activity *y*_*i*_ to a rotational mode would be to take *y*_*i*_ to be directly proportional, in the spirit of direct control, to a target angular velocity. Here we consider an alternative mechanism which allows for the generation of self-stabilizing attractors in the sensorimotor loop. For this purpose we use two steam-locomotive-like actuators that allow to translate the finite range of neural activity into a rotational model.

**Figure 1:**
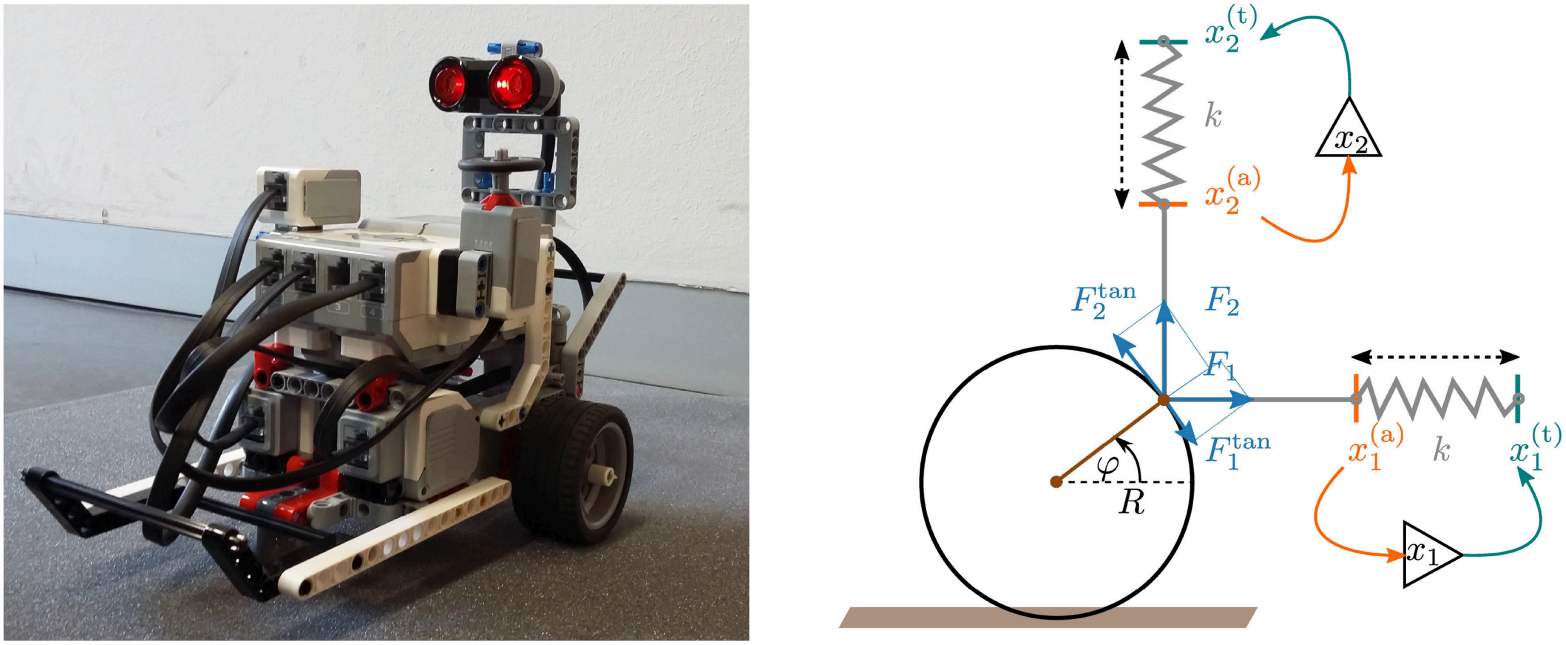
**Left**: The two-wheeled real robot constructed with the LEGO Mindstorms package. The active wheels are controlled by independent motors. A third passive wheel (the spherical shaped metallic wheel below the robots) keeps the body horizontal. **Right**: A sketch of a wheel with two perpendicular actuators (click for movie). A spring with spring constant *k* pulls the rod (in gray) toward the target position $x_{i}^{\left(t\right)}$ (green). The target position $x_{i}^{\left(t\right)}$ is determined by the output of a controlling neuron, as described by Equation (6), which receives in turn the actual position $x_{i}^{\left(a\right)}$ (orange) of the wheel as an input. Compare Equation (5). The final torque acting on the wheel is given by the sum of the tangential components of the spring forces *F*_1_ and *F*_2_, see Equation (8).

The two simulated actuators used for each wheel have a perpendicular alignment that allows for a continuous tracking. The respective transmission rods are fixed at one point of the perimeter of the wheel, as illustrated in Figure 1, being moved at the other end by ideal springs with constant *k*. The spring forces

(4) $$\begin{array}{l} F_{i}=k\left(x_{i}^{\text{(t)}}-x_{i}^{\text{(a)}}\right),\;i=1,2, \end{array}$$

are proportional to the distance $x_{i}^{\text{(t)}}-x_{i}^{\text{(a)}}$ between the normalized target position $x_{i}^{\text{(t)}}$ and the actual position $x_{i}^{\text{(a)}}$ of the wheel. The actual positions $x_{i}^{\text{(a)}}\in \left[-1,1\right]$ are determined in turn by the projections of the rotational angle φ of the wheel, measured respectively relative the horizontal and the vertical direction:

(5) $$\begin{array}{l} x_{1}^{\text{(a)}}=\cos\varphi ,\;x_{2}^{\text{(a)}}=\sin\varphi . \end{array}$$

The target positions $x_{i}^{\text{(t)}}\in \left(-1,1\right)$ are provided on the other side by the output of two independent rate-encoding neurons,

(6) $$\begin{array}{l} x_{i}^{\text{(t)}}=2y\left(x_{i}\right)-1,\;y\left(x\right)=\frac{1}{1+e^{-ax}}, \end{array}$$

which are characterized by a sigmoidal transfer function *y*(*x*) ∈ [0, 1] with slope *a*/4. The dynamics of the membrane potential *x*_*i*_ is driven in turn by the proprioceptual input $x_{i}^{\text{(a)}}$,

(7) $$\begin{array}{l} \tau {ẋ}_{i}=x_{i}^{\text{(a)}}-x_{i},\;i=1,2, \end{array}$$

where the internal time scale τ is of the order of a few hundred milliseconds. The total tangential force acting on the wheel is then the vectorial sum of the projections of individual spring forces (see Figure 1):

(8) $$\begin{array}{l} F^{\text{tan}}=F_{1}^{\text{tan}}+F_{2}^{\text{tan}}=F_{1}\sin\varphi -F_{2}\cos\varphi . \end{array}$$

The proposed controller translates hence the forth and back dynamics of the normalized neural activity *y* ∈ [0, 1] to a rotational motion for the wheel. For an animated illustration of the steam-locomotive controller see the Supplementary Material.

Note that a single actuator, e.g., a single horizontal transmission rod, would lead to a sinusoidal force $\propto \text{​ }F_{1}^{\text{tan}}$ vanishing at φ = 0 and φ = π. The combined force (8) is on the other hand always finite when two actuators with a perpendicular alignment are employed. See Figure 1 and the illustrating movie presented in the Supplementary Material.

### 2.3. LEGO mindstorms robot

To test the presented actuators we constructed robots with two active wheels using the LEGO Mindstorms Education Core Set (see the left picture of Figure 1). For more details about the experimental setup see the Supplementary Material. A third passive wheel keeps the body of the robot in a horizontal position. The active wheels are driven by motors that provide sensory feedback regarding the angle φ of the individual wheels. The working regime of the LEGO motors is finite, that is they respond to inputs $\tilde{M}\in \left[-{\tilde{M}}^{\text{max}},{\tilde{M}}^{\text{max}}\right]$. In order to comply with this constraint we mapped the simulated tangential force *F*^tan^, defined by Equation (8), via

(9) $$\begin{array}{l} \tilde{M}={\tilde{M}}^{\text{max}}\tanh\left(F^{\text{tan}}\right) \end{array}$$

to the motor signal $\tilde{M}$. For an elastic response we used typical relative motor commands of the order of $M=\tilde{M}/{\tilde{M}}^{\text{max}}\in \left[-0.7,0.7\right]$. Absolute time was measured at the start of every control loop and compared with the last time the control loop was called. The such determined time difference Δ*t* between two successive instances of the control loop was used for solving (7) via a straightforward Euler integration. On the average we had Δ*t* ≈ 40ms.

The motor stalls for low motor powers, viz when $|\tilde{M}|\leq 0.1{\tilde{M}}^{\text{max}}$, as a consequence of the internal friction of the gearing. A minimal torque is hence required for the robot to start moving. The overarching dynamical system, describing the body, the internal controller and the interactions between body and environment, allows for the generation of self-organized attractors (Sándor et al., 2015) that correspond to different motion patterns, the motor primitives (Ijspeert et al., 2002).

## 3. Results

### 3.1. Self-organized attractors as motor primitives

**Figure 2:**
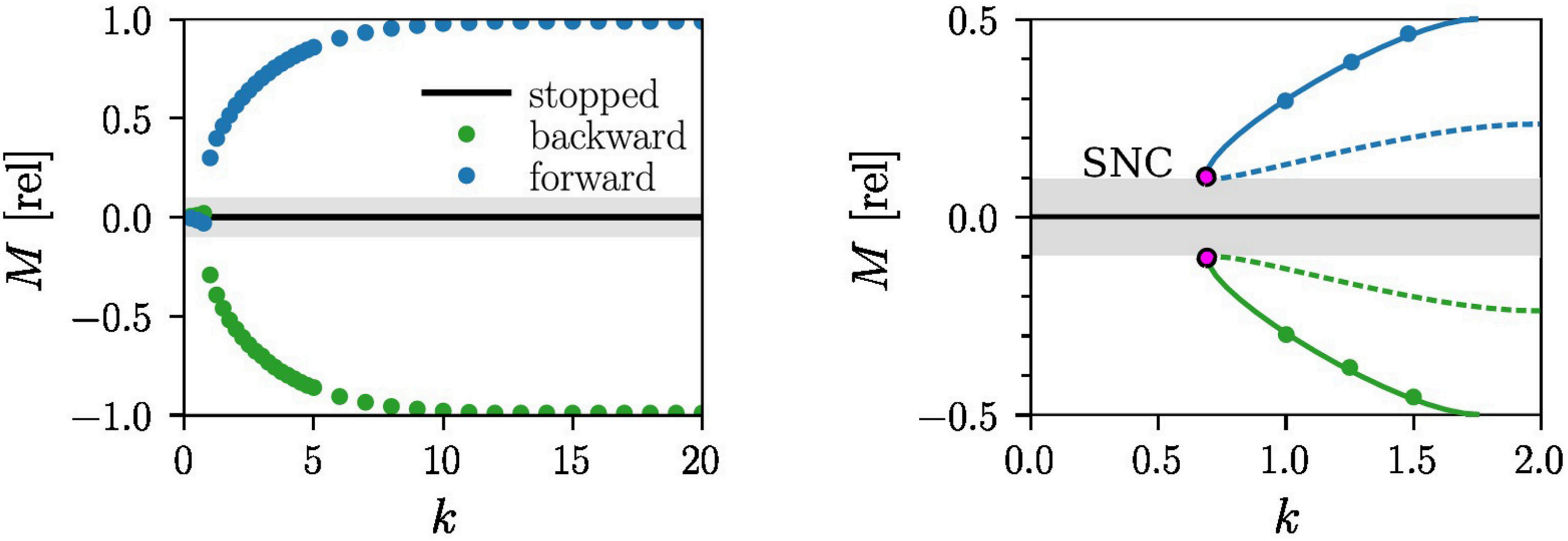
Motion primitives corresponding to non-moving and to forward and backward limit-cycle locomotion. Note that the motor stalls for |*M*| < *M*^thr^ ≈ 0.1 (gray area), viz when the torque *M* is unable to overcome the internal friction. **Left**: The measured output torque *M* for *a* = 4 and τ = 250ms as a function of the spring constant *k*. The measurements are performed after the robot settles in the forward (blue) or in the backward (green) attractor. For small *k* the robot stops moving and the tangential force vanishes. **Right**: An enlargement of the region *k* ∈ [0, 2] showing the measurements (blue/green dots) and the corresponding stable limit cycle (blue/green lines). A saddle-node bifurcation of limit-cycles (SNC) is likely to occur when the torque is counteracted by the internal friction. The resulting unstable limit cycles (dashed blue/green lines) are shown.

In the normal mode the robot disposes, as shown in Figure 2, of three possible states: stopped, forward and backward moving. For the forward and backward limit cycle locomotion the torque acting on the wheels is quasi-stationary, an observation that is consistent with the analytic treatment detailed out in section 4. Additionally, the robot may also rotate around its own axis, which happens when the two active wheels turn in opposite directions or when only one of the two wheels turns.

**Figure 3:**
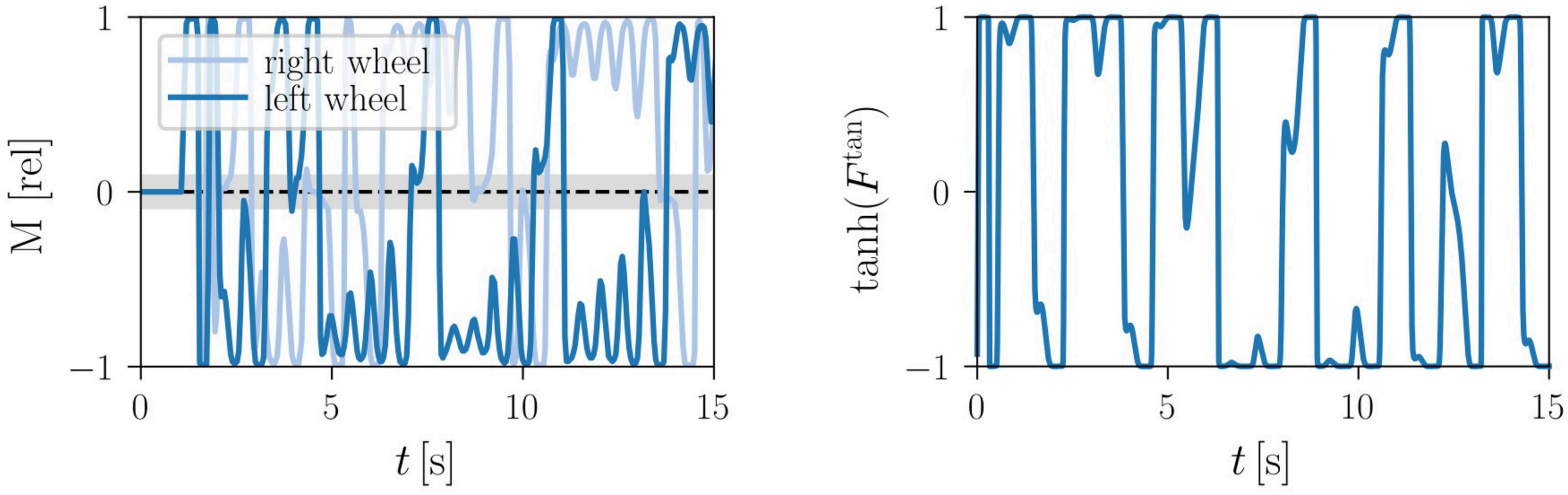
Time series of the motor torques (9) in the chaotic mode, in relative units, for *a* = 4, τ = 1, 000ms and *k* = 15. **Left**: For the LEGO robot (click for movie). The light gray shaded area indicates the |*M*| < *M*^thr^ ≈ 0.1 region where the motor stalls. **Right**: For the analytic model (13), with *F*^tan^ given by the torque on right-hand side of $I\overset{\cdot }{\omega }$. Here we took *f* = 0.5 for the friction and *I* = 0.05 for the moment of inertia.

The measured speed of the robot in the forward and backward moving modes is *v* = 0.35m/s for *k* = 8, *a* = 4, and τ = 250ms, a setting that makes use of about 80–90% of the maximal power of the motor. In addition to the basic limit cycle attractors one finds for *a* = 4, *k* = 15, and τ = 1, 000ms a chaotic attractor, for which the motors switch irregularly between the destabilized fundamental modes of the individual wheels, as illustrated in Figure 3. For a video of the chaotic dynamics of the robot see the Supplementary Material. We did not attempt, for the case of the LEGO robot, to fully map the set of parameters for which a stable chaotic attractor exists, e.g., by evaluating the Lyapunov exponents in the context of navigation (Harter and Kozma, 2005). We note that chaotic attractors have been shown to be useful in the design and construction of spatial navigation models (Voicu et al., 2004).

### 3.2. Kick control for embodied robots

The presence of coexisting attractors, viz of multistability (Pisarchik and Feudel, 2014), allows to switch between the basic modes without the need to modify the internal parameters of the system for the entire locomotion. The transitions between the individual attractors may be induced by external physical stimuli, such as collisions with other robots or with the environment (Martin et al., 2016).

**Figure 4:**
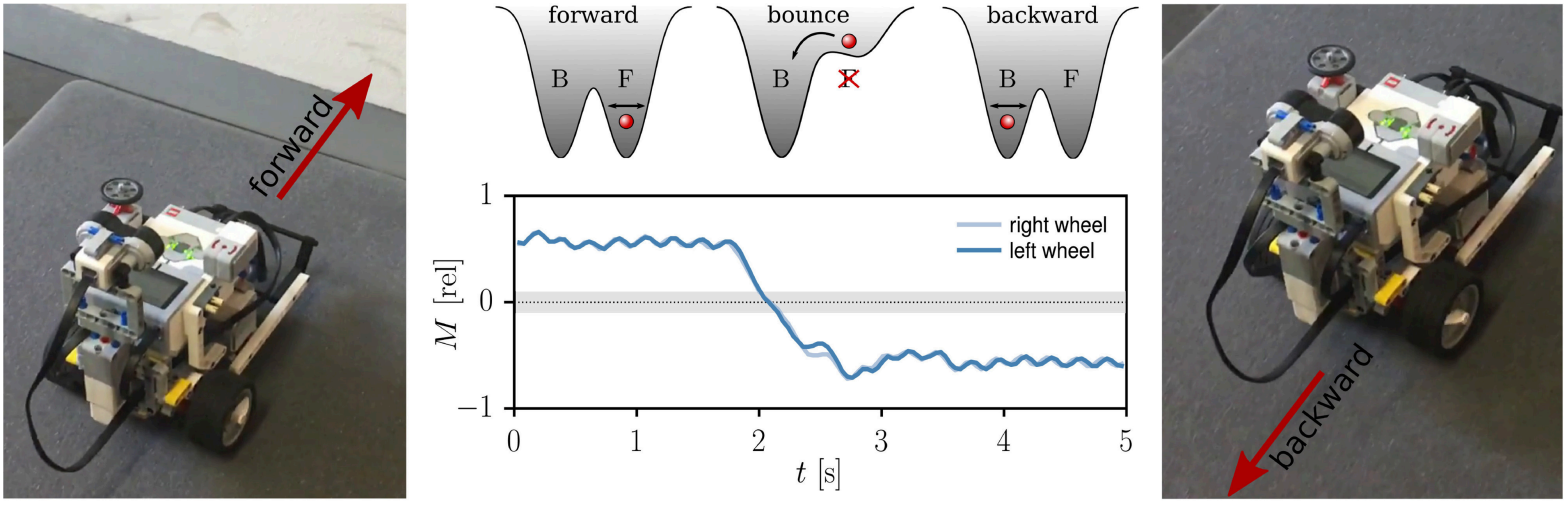
Collision induced switch of attractors. **Middle**: The time series of the relative torque *M* acting on the wheels for τ = 250ms, *k* = 2 and *a* = 4. The gray shaded region indicates the minimal torque needed to start the motor, *M*^thr^ = 0.1. The superimposed sketches illustrate a double-well potential with the minima corresponding to two coexisting attractors. The phase point (red ball) stays around the minima even in the presence of noise or small oscillations. The system is located in the backward attractor (B) when the robot collides with a wall and the forward attractor (F) is destabilized. The total torque *M* changes consequently its sign. **Right, Left**: The Lego robot before and after colliding with the wall (click for movie).

A robot initialized in the forward moving mode is able to reverse direction when bouncing off a wall placed perpendicularly to the direction of locomotion, as illustrated in Figure 4. The reversal of direction is performed in this case autonomously, that is in absence of any additional control signals. It occurs because the forward mode gets destabilized for the duration of the collision, whereas the basin of attraction of the backward mode expands correspondingly. The flow in phase space is then drawn toward the backward attractor, where it stays after the forward attractor reemerges upon pulling away from the wall. Autonomous switching between forward and backward modes when colliding with obstacles occurs robustly, as we demonstrated by a series of experiments on rough surfaces (see Supplementary Video 3).

An alternative possibility to generate switches between coexisting attractors is to kick the phase point of the dynamical system to the basin of attraction of another attractor, termed here as kick control. This may be realized by applying short duration input stimuli. We present here three intuitive mechanisms, which may be classified, as discussed in section 2.1, as “frozen” and “quenched” kick control.

#### 3.2.1. Frozen kick control

**Figure 5:**
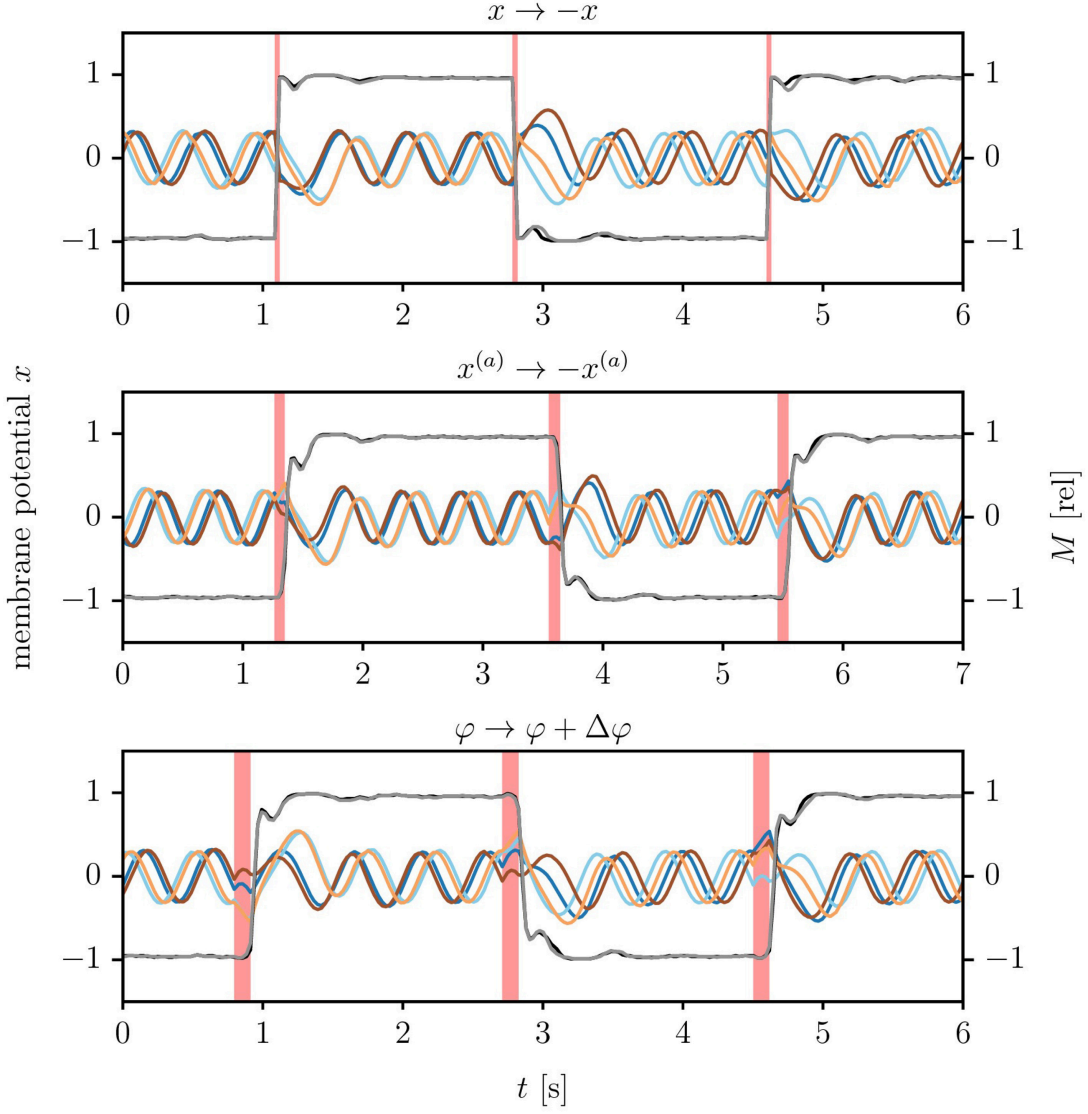
Kick controlling the LEGO Mindstorms robot. Shown are the time series of the membrane potentials (brown/orange and blue/cyan lines for *x*_1_/*x*_2_ of the left/right wheel), together with the normalized motor control $M=\tilde{M}/{\tilde{M}}^{max}$ (black/gray lines for left/right wheel). The parameters τ = 250ms, *k* = 8 and *a* = 4 lead to an angular frequency of ω/(2π) ≈ 2Hz. **Top**: Inverting all membrane potentials *x*_*i*_, compare (10), with the times indicated by the vertical red lines (click for movie). **Middle**: Inverting all actual positions $x_{i}^{\left(a\right)}$, see (11), for three ticks of the updating cycle (roughly 90 ms, as indicated by the red vertical bars). **Bottom**: Adding a phase shift of Δφ = ±3π/4 to the measured angle of the wheels, see (12). The length of the control signal is here 4 ticks, corresponding to 120 ms. Note that the time to settle in the reverse limit cycle exceeds the duration of the kick signal. The relative phase of the left and the right wheel increases considerably after the second reversal.

A reliable direction reversal may be induced by inverting membrane potentials via

(10) $$\begin{array}{l} x_{1}\;\to \;-x_{1},\;x_{2}\;\to \;-x_{2}, \end{array}$$

at given time. Equation (10) induces an instantaneous change of the internal variables that does not affect the parameters of the one-neuron controller. It corresponds therefore, as defined in section 2.1, to frozen kick control. The respective time-series of the membrane potentials are shown in Figure 5. The reversal of the direction occurs fast, depending however on the state the actuator was in when the membrane potentials were inverted. It is furthermore noticeable, in particular following the second kick signal, that it may take half a second or more to fully settle into the reversed attractor. A relative phase slip may be induced in addition in between the two wheels, which are mechanically not precisely identical. The here considered variant (2) of frozen kick control is furthermore 100% reliable in direction reversal tests (for a demonstration see Supplementary Video 4). This is due to the fact that the sign flip of internal variables *x*_1, 2_ leads instantaneously to a motor torque of opposite direction, compare Equations (4, 8).

#### 3.2.2. Quenched kick control

We considered two variants of quenched kick control. For the first variant one substitutes the actual wheel positions $x_{1,2}^{\left(a\right)}$ by

(11) $$\begin{array}{l} \begin{array}{ccc} x_{1}^{\left(a\right)}\; & \to & \left(1-2\beta \right)\cos\left(\varphi \right)\\ x_{2}^{\left(a\right)}\; & \to & \left(1-2\beta \right)\sin\left(\varphi \right) \end{array},\;\beta =\{\begin{array}{cc} 0 & \text{(control off)}\\ 1 & \text{(control on)} \end{array}\;. \end{array}$$

The new parameter β = β(*t*) is turned on for a finite control period Δ*t*, as illustrated in Figure 5. This control procedure mimics (10) in the sense that the reversal of the membrane potentials is not achieved by a direct kick in the phase space of internal variables, but by a change of parameters. Compare Equation (7).

**Figure 6:**
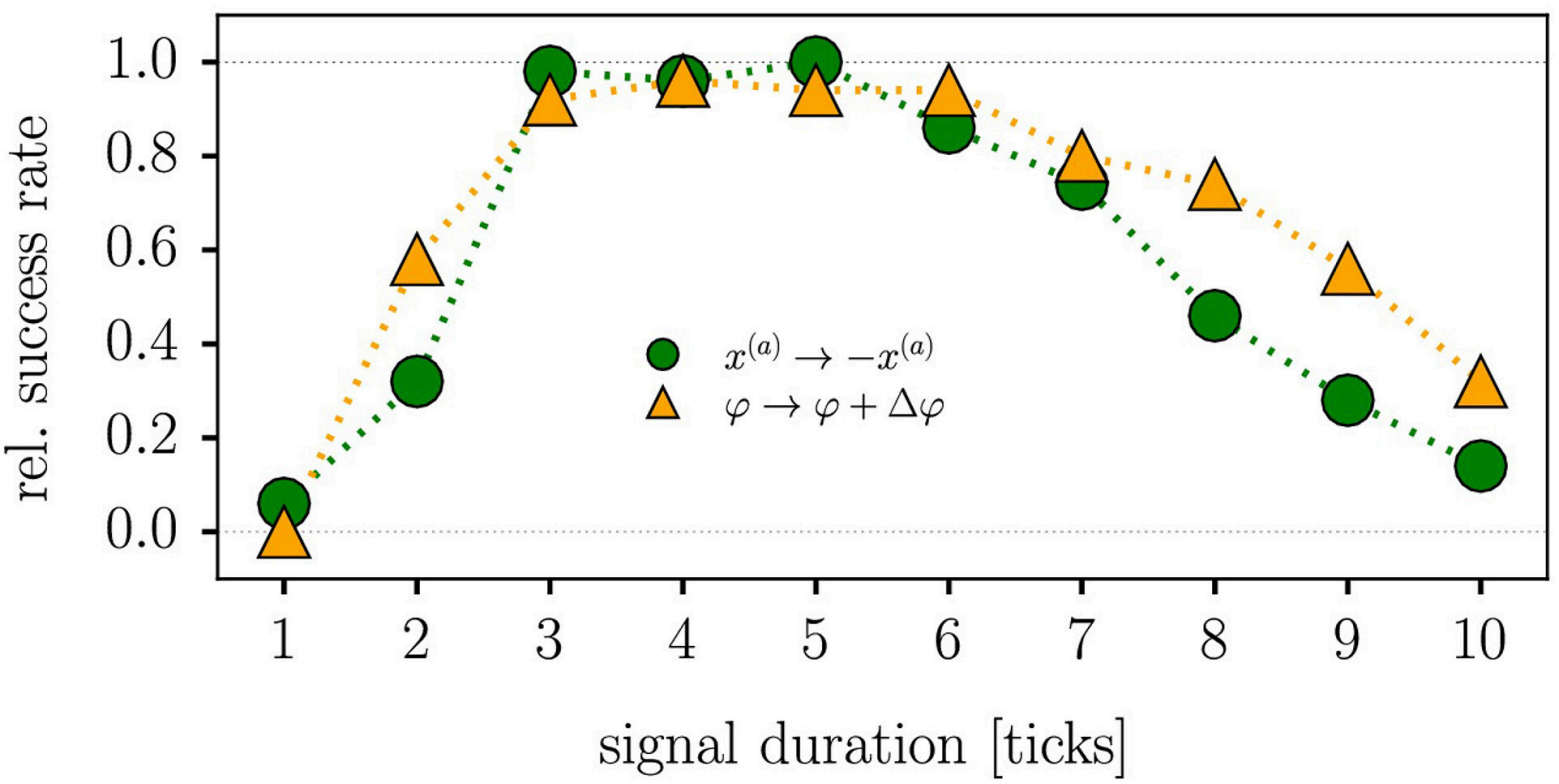
Reliability of quenched kick control procedures for the LEGO Mindstorms robot in terms of the relative success rate when testing direction reversal. The parameters are τ = 250ms, *k* = 8 and *a* = 4, with each data point corresponding to the average of 50 trials. The duration Δ*t* of the control signal is given in terms of ticks, with a tick corresponding to the period of the control cycle of the Lego robot (about 30 ms). Maximal reliability is achieved for 3–5 ticks (90–120 ms), both for reversing the actual position *x*^(*a*)^ [compare (11), green circles] and when adding a phase-shift of Δφ = ±3π/4 to the angle of the wheel [as defined by (12), orange triangles]. A positive Δφ induces here a transition from forward to backward locomotion (reversely for negative Δφ).

Real-world robots come with a control cycle that discretizes time. We consequently measure the time Δ*t* during which β(*t*) is active in terms of control cycles (ticks). In Figure 6, we present the results of an experiment testing the reliability of (11), that is for the probability that the robot reverses direction for a given Δ*t*. For the time-series shown in Figure 5 the robot received a kick signal for three ticks, viz for about 90 ms.

For an alternative type of quenched kick control we consider with

(12) $$\begin{array}{l} \begin{array}{ccc} x_{1}^{\left(a\right)}\; & \to & \cos\left(\varphi +\gamma \Delta \varphi \right)\\ x_{2}^{\left(a\right)}\; & \to & \sin\left(\varphi +\gamma \Delta \varphi \right) \end{array},\;\gamma =\{\begin{array}{cc} 0 & \text{(control off)}\\ 1 & \text{(control on)} \end{array} \end{array}$$

a shift in the sensory value of the angle of the robot. The corresponding reliability statistics are also shown in Figure 6. Both types of quenched kick control, as defined by Equations (11, 12), need to be applied for 3–5 control ticks, corresponding to 90–150 ms, for the robot to turn direction. For the experiment presented in Figure 5 we used 4 ticks when kicking the robot via (12).

### 3.3. Self-organized train of cars

**Figure 7:**
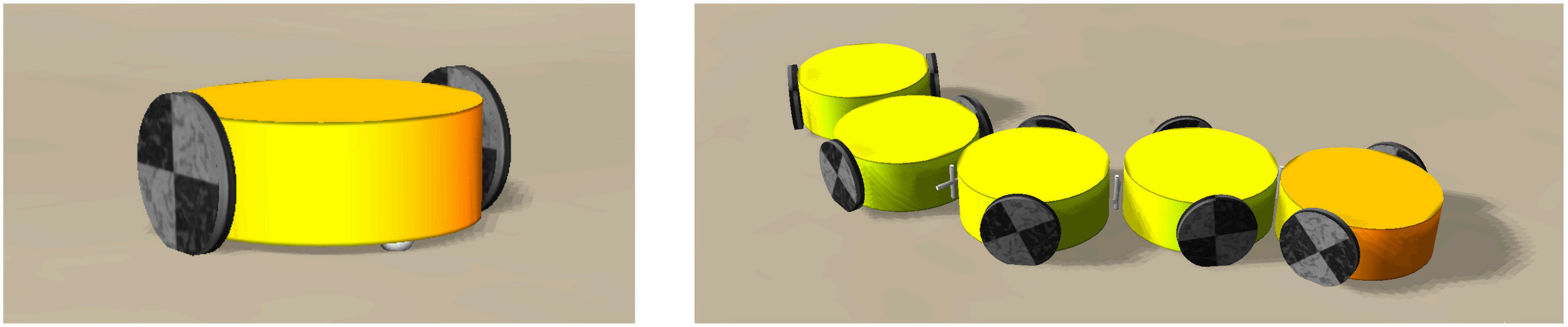
Snapshots from the LPZRobots simulations environment (Der and Martius, 2012) of two-wheeled car-like and train-like robots. **Left**: The two active wheels (black) are controlled as described in section 2.2. The passive support wheel (white) below the body (yellow) prevents the car from tipping over (click for movie). **Right**: A train robot created by connecting cars via passive torsion springs (click for movie).

We used the LPZRobots simulations environment (Der and Martius, 2012) to simulate robots with two actuated wheels, as illustrated in Figure 7. The individual robots have a body mass of 0.1kg, wheel mass of 0.05kg, wheel radius of 3cm, body radius of 10cm and body height of 5cm. The dimensionless friction coefficient is 0.1. The cars are controlled as described in section 2.2, but this time only a single simulated transmission rod is employed. This is possible, as the motor of the simulated robots transits to an idle state in the absence of an input signal.

The single two-wheel car follows intricate non-holonomic trajectories when put in an environment containing confining slopes. We also constructed trains composed of two-wheel cars coupled passively via torsion springs. All 10 wheels, for the case of the five-car train shown in Figure 7, are independent. One observes that the ten wheels coordinate their rotations speed and direction, reacting in a coordinated manner upon encountering objects in structured environment. The resulting locomotion of the train is a prime example of a self-organizing process (see Supplementary Videos 5, 6).

## 4. Analytic modeling

The fundamental attractor of the individual wheels may be studied analytically when lumping the feedback of the environment into a single equation of motion that contains friction in terms of a friction force ∝​fω. The resulting dynamical system is then

(13) $$\begin{array}{l} \tau {\dot{x}}_{1}=\cos\varphi -x_{1}\\ \tau {\dot{x}}_{2}=\sin\varphi -x_{2}\\ \;\dot{\varphi }=\omega \\ \;I\dot{\omega }=k(2y(x_{1})-1)\sin\varphi -k(2y(x_{2})-1)\cos\varphi -f\omega , \end{array}$$

where ω denotes the angular velocity and φ the angle of the wheel. The membrane potential of the horizontal and vertical controllers are, as illustrated in Figure 1, *x*_1_ and *x*_2_. The moment of inertia of the wheel is proportional to *I*, the spring constant by *k*, the friction coefficient by *f*, the time constant of the membrane potentials by τ and the transfer function *y*(*x*) of the controlling neurons by the sigmoidal *y*(*x*) = 1/(1 + exp(−*ax*)).

Note that *x*_1_ and *x*_2_ are internal variables of the robot, whereas φ (and consequently also ω) corresponds to the physical angle of the wheel and therefore to an environmental variable. This is because the body of the robot, including the wheels, are, from the perspective of the neural circuitry, part of the environment. We also point out that the motor power needs to exceed a certain threshold for the LEGO Mindstorms actuators to become active, as explained in section 2.3. This feature goes however beyond Equation (13).

### 4.1. Stationary wheel

The system of four coupled differential equations (13), possesses eight trivial fixpoints, characterized by

(14) $$\begin{array}{l} \omega_{n}^{*}=0,\;\varphi_{n}^{*}=n\pi /4,\;n\in \left\{0,\ldots ,7\right\}\;, \end{array}$$

of which the odd multiples of π/4 are always unstable. The even multiplies of π/4 are on the other side stable/unstable for small and large ratios of *akτ*/(2*f*), respectively, as we will show further below. The two-wheeled robot can hence be in 4 × 4 = 16 non-moving states corresponding to the 16 combinations of stable fixpoints of Equation (14) of the left and right wheel.

### 4.2. Homoclinic route to locomotion

In order to understand the transition from the fixpoint solutions (14) to locomotion we use

(15) $$\begin{array}{l} \cos\varphi \left(t^{\prime }\right)\approx \cos\varphi \left(t\right)-\sin\varphi \left(t\right)\overset{\cdot }{\varphi }\left(t\right)\left(t^{\prime }-t\right)\\ \;=\cos\varphi \left(t\right)+\sin\varphi \left(t\right)\omega \left(t\right)\left(t-t^{\prime }\right)\;, \end{array}$$

which is valid for ωτ ≪ 1 and *t* − *t*′ ≪ τ, to expand the formal integral

(16) $$\begin{array}{l} x_{1}\left(t\right)=\frac{1}{\tau }\int_{-\infty }^{t}dt^{\prime }\cos\varphi \left(t^{\prime }\right)e^{-\left(t-t^{\prime }\right)/\tau }\approx \cos\varphi \left(t\right)\;+\;\omega \left(t\right)\tau \sin\varphi \left(t\right)\; \end{array}$$

of *x*_1_(*t*). An equivalent expansion may be derived for *x*_2_(*t*). One next expands the neural transfer functions occurring on the right-hand side of $\overset{\cdot }{\omega }$,

(17) $$\begin{array}{l} \begin{array}{ccc} y\left(x_{1}\right) & \approx & y\left(\cos\varphi \right)+ay\left(\cos\varphi \right)\left(1-y\left(\cos\varphi \right)\right)\omega \tau \sin\varphi \\ y\left(x_{2}\right) & \approx & y\left(\sin\varphi \right)-ay\left(\sin\varphi \right)\left(1-y\left(\sin\varphi \right)\right)\omega \tau \cos\varphi \end{array} \end{array}$$

for small ωτ. The equations of motion (13) then take the form

(18) $$\begin{array}{l} \overset{\cdot }{\varphi }=\omega ,\;I\overset{\cdot }{\omega }=F\left(\varphi \right)+\gamma \left(\varphi \right)\omega \;, \end{array}$$

where *F*(φ) is a mechanical force and γ(φ) the coefficient of an adaptive friction. The respective expressions are

(19) F=2k[y(cosφ)sinφ−y(sinφ)cosφ]+k[cosφ−sinφ]

and

(20) $$\begin{array}{l} \gamma =2ka\tau [y(\cos\varphi )(1-y(\cos\varphi ))\sin^{2}\varphi \\ \;+y(\sin\varphi )(1-y(\sin\varphi ))\cos^{2}\varphi ]-f. \end{array}$$

Within this approximation, one finds that γ(φ) is negative for all φ ∈ [0, 2π] when

(21) $$\begin{array}{l} k<k_{c},\;k_{c}=\frac{2f}{a\tau }\;. \end{array}$$

**Figure 8:**
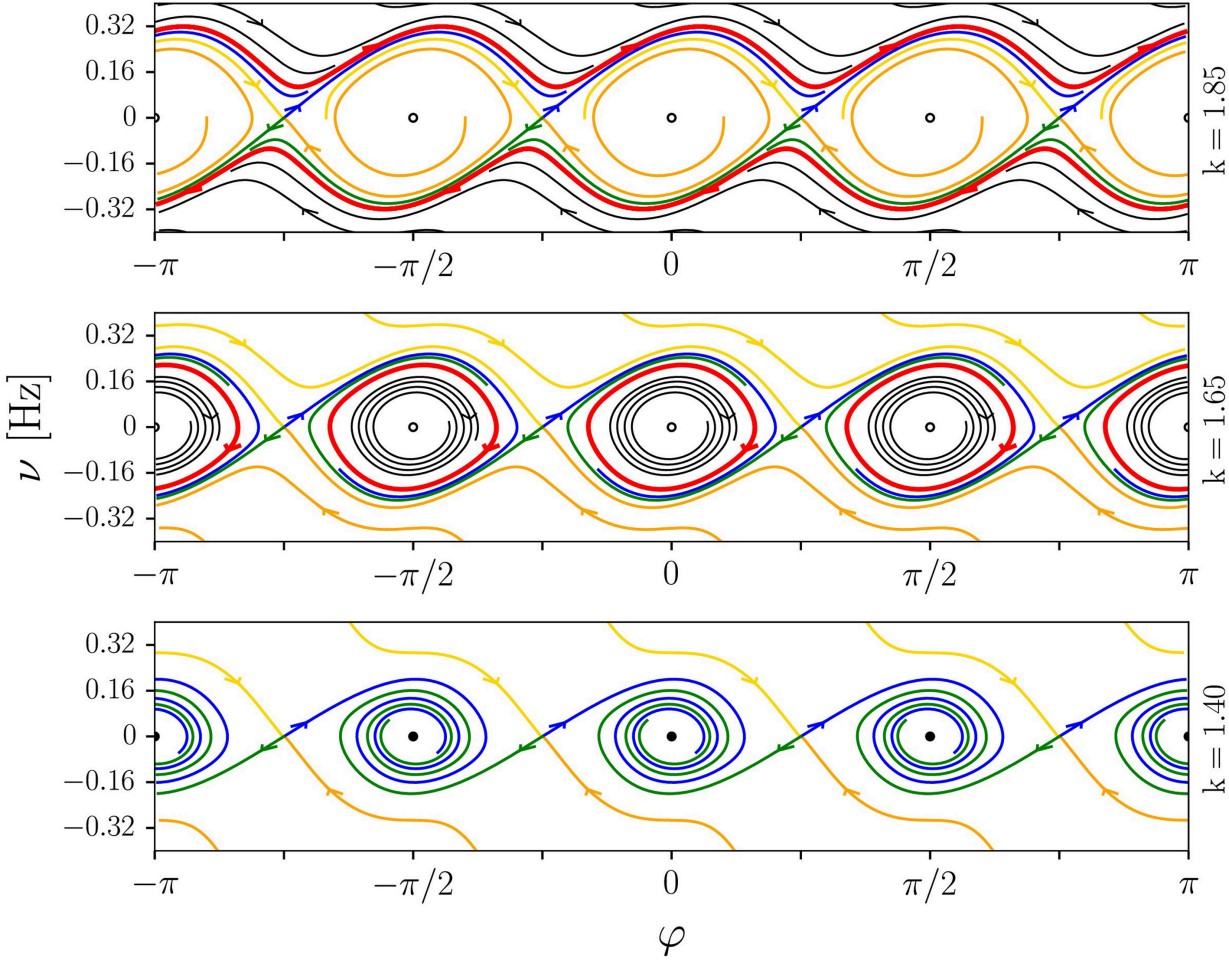
Illustration of the homoclinic route to locomotion discussed in section 4.2. The phase-space plots (φ, ν = ω/(2π)) are for the analytic model (13), with *a* = 4, τ = 0.25, *I* = 0.05, and *f* = 0.5. The spring constant is *k* = 1.85/1.65/1.4 (top/middle/bottom). The fixpoints located at odd multiples of π/4 are saddles. Shown are stable (yellow/orange) and unstable (blue/green) manifolds, limit cycles (red), stable and unstable foci (filled/open circles) and some selected generic trajectories (black). **Top**: Limit-cycle locomotion, with the stable orbit winding around φ ∈ [−π, π]. **Middle**: Forth-and-back rolling with the angle φ of the wheel being limited to a finite range around multiples of π/2. **Bottom**: Stationary states with φ → *nπ*/2.

The system is purely dissipative when γ < 0, which implies that the fixpoints $\varphi_{2n}^{*}=n\pi /2$ are stable for *k* < *k*_*c*_. Locomotion is next achieved via a two-step process, as illustrated in Figure 8, when increasing the spring constant *k* beyond *k*_*c*_.

– The fixpoints $\varphi_{2n}^{*}=n\pi /2$ undergo a supercritical Hopf bifurcation at *akτ* = 2*f*, viz when $\gamma \left(\varphi_{2n}^{*}\right)$ becomes positive. The angle φ of the wheel then oscillates around the previously stable fixpoint $\varphi_{2n}^{*}$, with a trajectory that corresponds to a periodic forth-and-back motion of the robot.
– The amplitude of the limit cycle in φ will reach eventually, when *k* is further increased, the saddles at $\varphi_{2n}^{*}=n\pi /2+\pi /4$. The limit cycle will then merge with the respective stable and unstable manifold of the saddle and undergo a Taken-Bogdanov-type (Gros, 2015) homoclinic bifurcation. Above this transition the four symmetry-related limit cycles around $\varphi_{2n}^{*}=n\pi /2$ merge into a large cycle. The wheel then performs complete rotations.

We note that an equivalent merging of symmetry related limit cycles across a global bifurcation has been observed in a study of prototype dynamical systems (Sándor and Gros, 2015).

### 4.3. Constant velocity approximation

The angular moment ω becomes nearly constant for *k* far above the infinite-period transition. The solution of Eqution (7) obtained in the limit *t* → ∞ is given for the case of a constant angular velocity as

(22) $$\begin{array}{l} x_{1}\left(t\right)=\frac{\cos\left(\omega t\right)+\omega \tau \sin\left(\omega t\right)}{1+\omega^{2}\tau^{2}},\;x_{2}\left(t\right)=\frac{\sin\left(\omega t\right)-\omega \tau \cos\left(\omega t\right)}{1+\omega^{2}\tau^{2}}, \end{array}$$

where we have used φ(*t*) = ω*t*. Assuming small amplitude oscillations for the membrane potential, *ax*_*i*_ ≪ 1, we can linearize the transfer function *y*(*x*) around *y*(0) = 1/2. The total tangential force *F*^tan^ defined by Equation (8) then becomes constant,

(23) $$\begin{array}{l} F^{\tan}=\frac{ak\omega \tau /2}{1+\omega^{2}\tau^{2}},\;\frac{ak\omega \tau /2}{1+\omega^{2}\tau^{2}}=f\omega ,\;\omega_{\pm }^{*}=\pm \sqrt{\frac{ak\tau -2f}{2f\tau^{2}}}\;, \end{array}$$

where the second equation corresponds to the balance between *F*^tan^ and the friction force *fω* in Equation (13). Locomotion vanishes in the constant-ω approximation for *k* < *k*_*c*_ = 2*f*/(*aτ*), viz at the critical spring constant *k*_*c*_ defined in (21).

The two symmetrical branches corresponding to the stable attractors of forward and backward motion can be seen in the experimentally constructed bifurcation diagram shown in the right panel of Figure 2. The internal friction forces of the motor induces in addition two symmetry-related saddle-node bifurcations of limit cycles, when reducing *k*, such that the torque *M* drops discontinuously to zero as *k* → *k*_c_. The internal threshold of real-world motors impacts the route to chaos hence qualitatively. Compare section 4.2.

### 4.4. Numerical and analytic phase diagram

**Figure 9:**
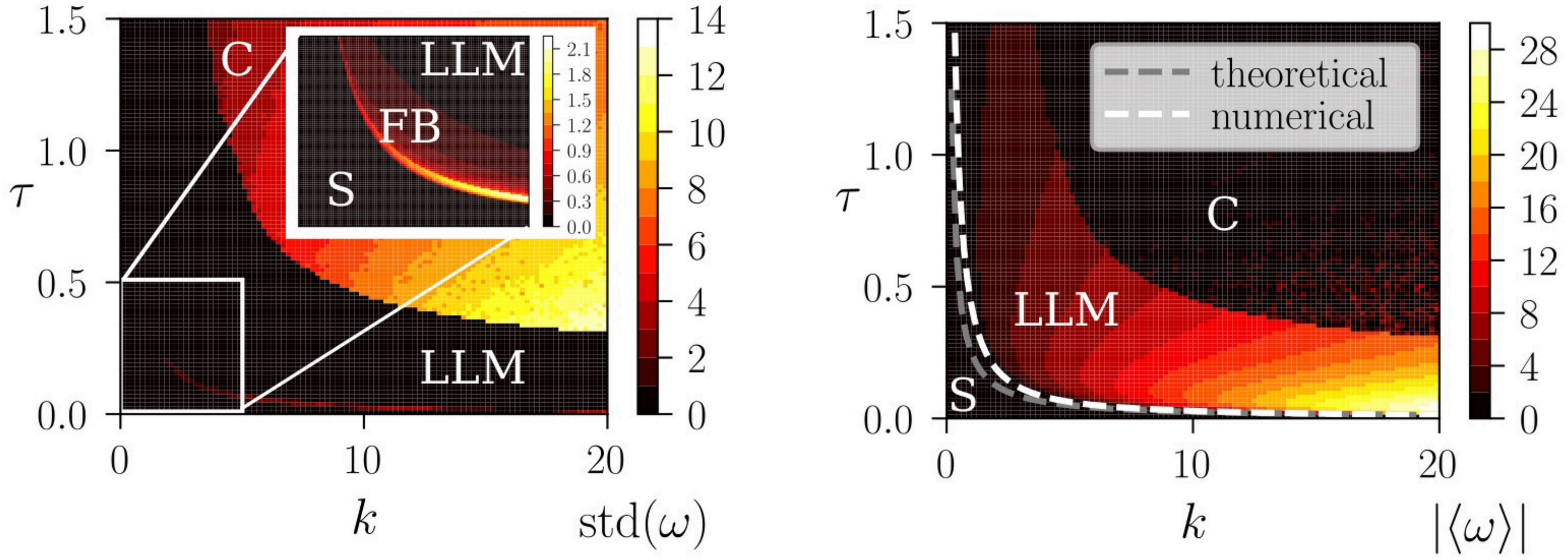
**Left**: The numerical phase diagram, as obtained by simulating (13) for *a* = 4, *I* = 0.05 and *f* = 0.5. Shown is the standard deviation $\text{std}\left(\omega \right)=\sqrt{\langle \omega^{2}\rangle -{\langle \omega \rangle }^{2}}$ of the angular frequency ω of the wheel (color coded), as averaged over time. Low values of std(ω) correspond to vanishing or near constant ω(*t*), as for the fixpoint solution (S) and for the limit-cycle locomotion (LLM). The transition to chaos (C) takes place through a crisis, with the route to locomotion occurring, as discussed in section 4.2, via an intermediate phase of forth-and-back rolling (FB). The inset enlarges the region *k* ∈ [0, 5] and τ ∈ [0, 0.5]. **Right**: The average angular frequency |〈ω〉| (color coded). The dashed lines correspond to the instability lines of the fixpoint solution, as obtained numerically (white) and analytically (gray), where the theoretical result is *k*_*c*_τ_*c*_ = 2*f*/*a*. See Equation (21).

In Figure 9, we present the phase diagram, as obtained by integrating (13) numerically. Four phases are found, a stationary fixpoint phase (S) at low values of *kτ*, a phase corresponding to forth-and-back (FB) motion, the limit-cycle locomotion phase (LLM) and, for large values of *kτ*, a region characterized by chaotic (C) locomotion. These phases may be characterized by the standard deviation $\text{std}\left(\omega \right)=\sqrt{\langle \omega^{2}\rangle -{\langle \omega \rangle }^{2}}$ of the angular frequency, which is elevated in the chaotic and in the FB phase, and small for LLM. The average angular frequency |〈ω〉| is in contrast highest for limit-cycle locomotion.

Also shown in Figure 9 is a comparison with the approximate estimate (21) of the transition between the stationary and the locomotive state, which is accurate when the transfer function *y*(*x*) can be linearized, viz in the limit of a vanishing FB phase. A typical time series of the motor torque within the chaotic phase is shown in the right panel of Figure 3.

## 5. Discussion

Robotic locomotion may be generated either via top-down control mechanisms or alternatively via self-organizing dynamical processes (Aguilar et al., 2016). In the first, more traditional approach, the “brain” of the agent is responsible for computing the control signals that drive the actuators, with error correction occurring through a high level evaluation of sensory measurements. The complexity of the control problem can however be reduced when robotic behavior is generated via self-organizing processes and local instabilities of the neural dynamics (Der and Martius, 2012).

Our work aims to reduce the theoretical and the computational constraints needed to design autonomous agents. Based on previous works on barrel- and sphere shaped robots (Sándor et al., 2015; Martin et al., 2016), we propose and study a novel actuator for wheeled robots. The actuator simulates the physics of the transmission rod used by classical steam engines, being controlled at the same time by only one or two rate-encoding neurons. Together with the wheel and with the proprioceptual environmental feedback, the controlling neurons form an overarching dynamical system that generates motion primitives in terms of stable attractors. The such produced self-organized fixpoints, limit cycles, and chaotic attractors, correspond to non-moving robots, to robots moving with constant speed and, respectively, to robots engaging in exploratory behaviors. All results are robust to noise present either in the environment or in the proprioceptual input stream (Martin et al., 2016).

A particular feature of the controller proposed here is that the direction of the movement, forward or backward, is selected by breaking time reversal symmetry. Studying two-wheeled robots we demonstrate that attractors corresponding to forward and backward motions coexist in the sensorimotor loop, allowing the robot to change direction autonomously when colliding with a wall. Switching between stable attractors can be achieved furthermore via a higher-order top-down control. Implementing frozen and quenched control signals, we are able to kick the robot reliably between distinct attractors, that is to kick the robot from one motor primitive into another motion primitive.

Examining in addition the bifurcation diagram leading from stationary states characterized by fixpoints in the phase space of the sensorimotor loop to limit-cycle locomotion, we find that two routes to locomotion exist: a single step process via a saddle node bifurcation of limit cycles, and a two-step scenario via a supercritical Hopf bifurcation followed by limit-cycle merging through distinct homoclinic bifurcations.

The here presented kick-control schemes demonstrate that simple impulse-like control signals are sufficient for creating complex behaviors whenever the motor primitives are given in terms of stable sensorimotor attractors. Hence one may speculate that kick control could also be used effectively in case of more complex robotic architectures, possibly in a combination with other types of control schemes (e.g., with the KA models; Harter and Kozma, 2005).

The concept of kick control also allows for a more general framework which is not necessarily neuro related. However, when it comes to the scalability of the proposed control scheme to robots with several dozens or even hundreds of degrees of freedoms and sensory channels, the most convenient underlying dynamical systems for the internal controller are adaptive neural networks with learning. The attractors there are then generalizations of the simple limit-cycle and chaotic attractors presented here, but the main idea of kicking the phase point from the basin of attraction of one attractor to another attraction domain remains the same.

Finally, we believe that the proposed model for generating attractors for locomoting robots and controlling their motion by kicking the phase point to their respective basins of attraction may also be used for teaching dynamical systems in advanced high school physics courses. The Lego robots allow for interactive demonstrations, e.g., in lab activities, of how attractors may be used in real-world applications, hence providing an intuitive understanding of the terminology and underlying phenomena.

## Author contributions

The manuscript was written by BS and CG. The experiments were conceived and designed by CG, BS, TK, MN, and LM. Lego experiments performed mainly by TK and MN. Simulations performed by LM. The data was analyzed by BS and CG with calculations proposed by MN, and plots produced by BS and TK.

### Conflict of interest statement

The authors declare that the research was conducted in the absence of any commercial or financial relationships that could be construed as a potential conflict of interest.
