# Supplementary material for: Kick Control: Using the Attracting States Arising Within the Sensorimotor Loop of Self-Organized Robots as Motor Primitives
Source: Front Neurorobot. 2018 Jul 11;12:40. doi: 10.3389/fnbot.2018.00040 (PMC6051224; doi:10.3389/fnbot.2018.00040)
Supplement: Supplementary file 7 [file Presentation_1.PDF]

Supplementary Material: Kick control: using the attracting states arising within the sensorimotor loop of self-organized robots as motor primitives

1 SUPPLEMENTARY VIDEOS

Here we provide supplementary videos to the different dynamical behaviors and control sequences discussed in the main paper.

1.1 Steam-locomotive actuators

The wheels of the robot are driven by regulating in real time the torque of the internal motors. The actual value of the torque is determined by the vectorial sum of the spring forces F_1 and F_2 (see Eq. (4) in the main paper) generated by the perpendicular actuators. The resulting tangential component of the spring forces is quasi-constant during each control cycle as illustrated by Supplementary Video 1. For the analytical proof see Sec. 4.2 of the article.

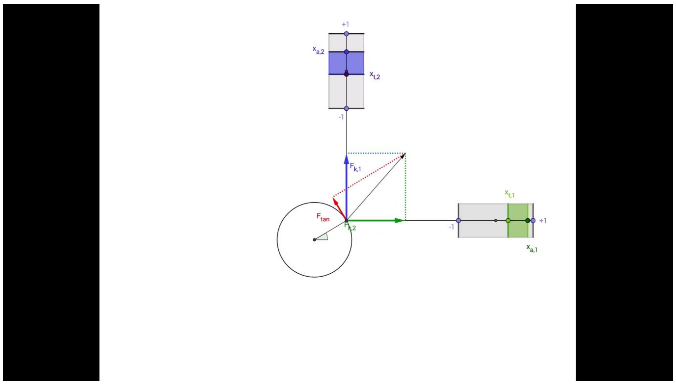

Supplementary Video 1. Steam-locomotive-like actuators driving one wheel of the robot. The two perpendicular actuators, akin to the transmission rods used for driving steam-locomotive wheels, are acting simultaneously on wheel through the spring forces F_1 and F_2 (blue and green arrows, respectively), compare the right sketch of Fig. 1 from the paper. The sum of the resulting tangential forces is quasi-constant (red arrow) leading to a constant speed locomotion. See: Video 1.mp4.

1.2 Experimental setup and self-organized attractors

The experimental setup used for testing the proposed control scheme consists of a two-wheeled Lego Mindstroms robot, constructed according to the official Lego Mindstorms Education ev3 Core Set manual, with two active wheels driven by two independent servo motors. The robot is equipped with a third sphere-like passive wheel as well, to keep the body of the robot balanced (see Supplementary Video 2). For programming the Lego robot we installed the ev3dev Debian-based Linux operating system (Hlubek et al., 2017), and used together with the Python language bindings for ev3dev (Hempel, 2015). The motor torques of the individual wheels, calculated as in Eq. (9), are then controlled via the high-level interface-library functions.

Due to the closed sensorimotor loop the resulting behavioral patterns correspond to self-organized attractors in the combined phase space of the controller, body and environment. For the two-wheeled robot used for the experiments we find three attractor types:

1. stable fixpoint attractors corresponding to a non-moving mode;
2. coexisting limit-cycle attractors allowing for constant-speed forward and backward locomotion;
3. chaotic attractors leading to exploratory dynamics.

The dynamical behavior generated during the chaotic motion is shown in Supplementary Video 2.

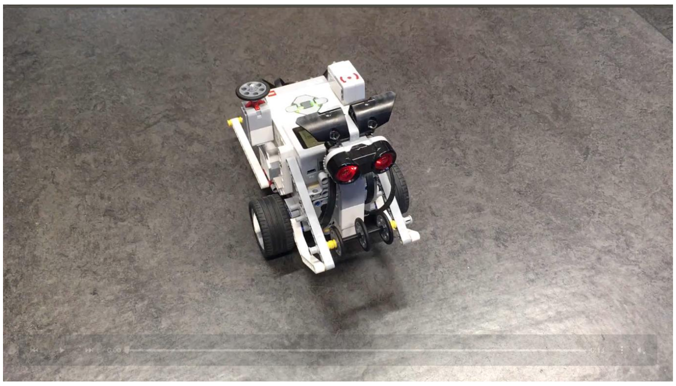

Supplementary Video 2. Chaotic dynamics of the two-wheeled Lego Mindstorms robot for the parameters: $a = 4$, $k = 15$ and $\tau = 1000\,\mathrm{ms}$. The corresponding time-series of relative motor torques are shown in Fig. 3 of the main paper. See: Video 2.mp4.

1.3 Collision induced direction reversal

The coexisting limit-cycle attractors of forward and backward moving are exploited by the robot upon colliding with a non-movable obstacle. At the moment of hitting a wall for example, the attractor of forward locomotion disappears and the flow in phase space is attracted towards the limit cycle with opposite locomotion direction (see Supplementary Video 3.). We emphasize that this direction reversal process is completely autonomous, and that the robot does not make use of any distance sensors upon approaching the wall. The only sensors involved in the direction reversal are proprioceptual, measuring the actual angle of the wheel's position.

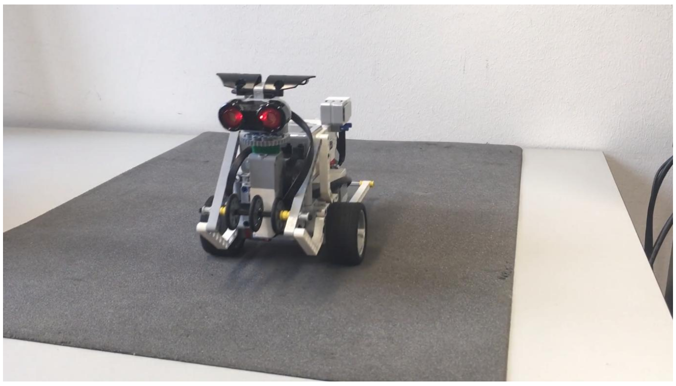

Supplementary Video 3. Collision induced switch of attractors. The robot is initialized in the basin of attraction of forward motion. After bouncing back from the wall it switches to backward locomotion. See: Video 3.mp4.

The collision induced switch of attractors can also be interpreted in the framework of kick control. When hitting the wall, the parameters characterizing the environment $\mathbf{P_E}$, compare Eq. (1), undergo an abrupt change, being restored to their original value after the robot moves away from the the obstacle.

1.4 Kick control schemes

An example of kick-controlled sequence of forward and backward locomotion modes, corresponding to the top panel of Fig. 5 is shown by Supplementary Video 4. The control signal, kicking the phase point from the forward to the backward attractor and vice-versa, is determined merely by the time-moments of the kicks, no other information is required.

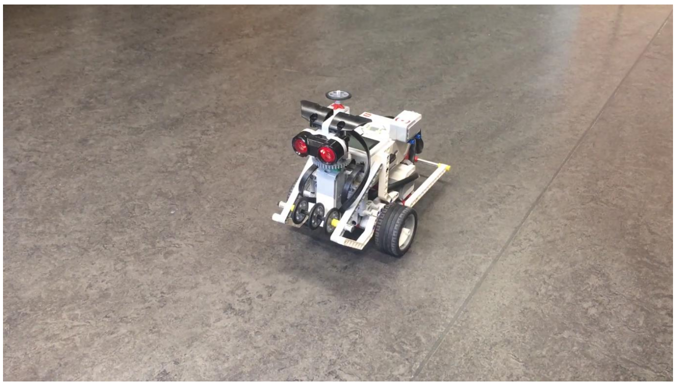

Supplementary Video 4. Kick-control test using the control scheme defined by the sign flip of membrane potential values, compare Eq. (11). The instantaneous control signals allow for a reliable direction reversal. The used parameters are: $\tau = 250\,\mathrm{ms}$, $a = 4$, $k = 8$. See: Video 4.mp4.

1.5 Simulated robots in complex environments

The steam-locomotive-like actuator controller has also been tested in a using the LpzRobots simulation software (Der and Martius, 2012). The two-wheeled car robot shows qualitatively similar behavior to the Lego robot's dynamics, tested with direction reversal experiments when colliding with a hard wall. A complex dynamics results also when the robot is placed in an arena surrounded by slopes (see Supplementary Video 5).

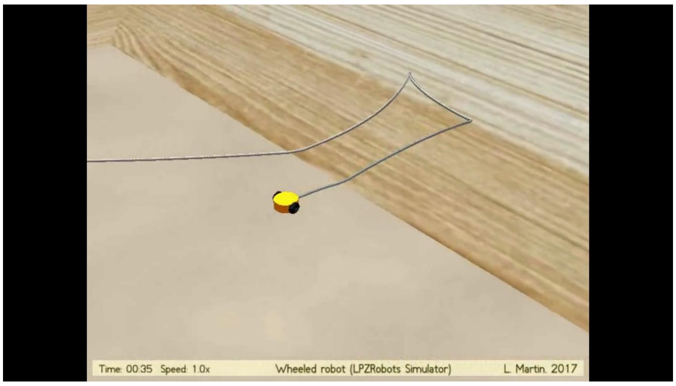

Supplementary Video 5. Video of the two-wheeled car robot in an arena with tilted walls, captured by using the LPZRobots simulation environment. The trace of the robot on the ground floor is illustrated by the tick white line. See: Video 5.mp4.

We have also tested the actuators in more complex robots, composed of five identical two-wheeled car elements, which are connected by passive torsion springs to keep the body of the ”snake robot” straight. The locomotion here is in turn generated via the synchronization of individual wheels, which tend to self-organize in a phase-locked state for moving along a straight-line. When colliding with obstacles the synchrony is broken temporarily. See Supplementary Video 6.

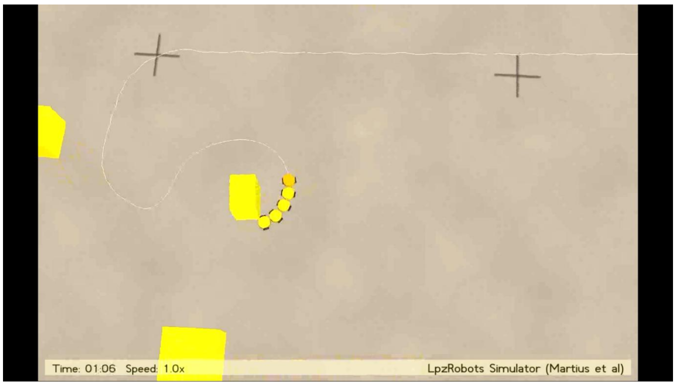

Supplementary Video 6. Train robot composed of five two-wheeled car elements in an environment with movable blocks (yellow). The robot shows playful behavior, pushing the blocks away then changing the direction of locomotion. See: Video 6.mp4.

REFERENCES

Der, R. and Martius, G. (2012). *The Playful Machine: Theoretical Foundation and Practical Realization of Self-Organizing Robots*, vol. 15 (Springer Science & Business Media)

[Dataset] Hempel, R. (2015). ev3-lang-python: Python language bindings for ev3dev. `https://github.com/ev3dev/ev3dev-lang-python`

[Dataset] Hlubek, C., Kortschak, D., Lechner, D., and French, R. (2017). ev3dev: a debian linux-based operating system. `http://www.ev3dev.org/`
